# Efficient targeting of HIF-1α mediated by YC-1 and PX-12 encapsulated niosomes: potential application in colon cancer therapy

**DOI:** 10.1186/s13036-023-00375-3

**Published:** 2023-09-25

**Authors:** Azar Bakand, Sevil Vaghefi Moghaddam, Maryam Naseroleslami, Helder André, Neda Mousavi-Niri, Effat Alizadeh

**Affiliations:** 1 Department of Medical Biotechnology, Faculty of Advanced Medical Sciences, Tabriz University of Medical Sciences, Tabriz, Iran; 2 Clinical Research Development, Unit of Tabriz Valiasr Hospital, Tabriz University of Medical Sciences, Tabriz, Iran; 3 Department of Cellular and Molecular Biology, Faculty of Advanced Science and Technology, Tehran Medical Sciences, Islamic Azad University, Tehran, Iran; 4 Department of Clinical Neuroscience, St. Erik Eye Hospital, Karolinska Institute, 11282 Stockholm, Sweden; 5 Department of Biotechnology, Faculty of Advanced Science and Technology, Tehran Medical Sciences, Islamic Azad University, Tehran, Iran

**Keywords:** Colon cancer, Drug delivery, Niosome, HIF-1α, YC-1, PX-12, Targeting

## Abstract

A number of molecular biofactors have been documented in pathogenesis and poor prognosis of colorectal cancer (CRC). Among them, the Hypoxia-Inducible Factor (HIF-1a) is frequently reported to become over-expressed, and its targeting could restrict and control a variety of essential hallmarks of CRC. Niosomes are innovative drug delivery vehicles with the encapsulating capacity for co-loading both hydrophilic and hydrophobic drugs at the same time. Also, they can enhance the local accumulation while minimizing the dose and side effects of drugs. YC-1 and PX-12 are two inhibitors of HIF-1a. The purpose of this work was to synthesize dual-loaded YC-1 and PX-12 niosomes to efficiently target HIF-1α in CRC, HT-29 cells. The niosomes were prepared by the thin-film hydration method, then the niosomal formulation of YC-1 and PX-12 (NIO/PX-YC) was developed and optimized by the central composition method (CCD) using the Box-Behnken design in terms of size, polydispersity index (PDI), entrapment efficiency (EE). Also, they are characterized by DLS, FESEM, and TEM microscopy, as well as FTIR spectroscopy. Additionally, entrapment efficiency, in vitro drug release kinetics, and stability were assessed. Cytotoxicity, apoptosis, and cell cycle studies were performed after the treatment of HT-29 cells with NIO/PX-YC. The expression of HIF-1αat both mRNA and protein levels were studied after NIO/PX-YC treatment. The prepared NIO/PX-YC showed a mean particle size of 185 nm with a zeta potential of about-7.10 mv and a spherical morphology. Also, PX-12 and YC-1 represented the entrapment efficiency of about %78 and %91, respectively, with a sustainable and controllable release. The greater effect of NIO/PX-YC than the free state of PX-YC on the cell survival rate, cell apoptosis, and HIF-1α gene/protein expression were detected (*p* < 0.05). In conclusion, dual loading of niosomes with YC-1 and PX-12 enhanced the effect of drugs on HIF-1α inhibition, thus boosting their anticancer effects.

**Graphical Abstract:**

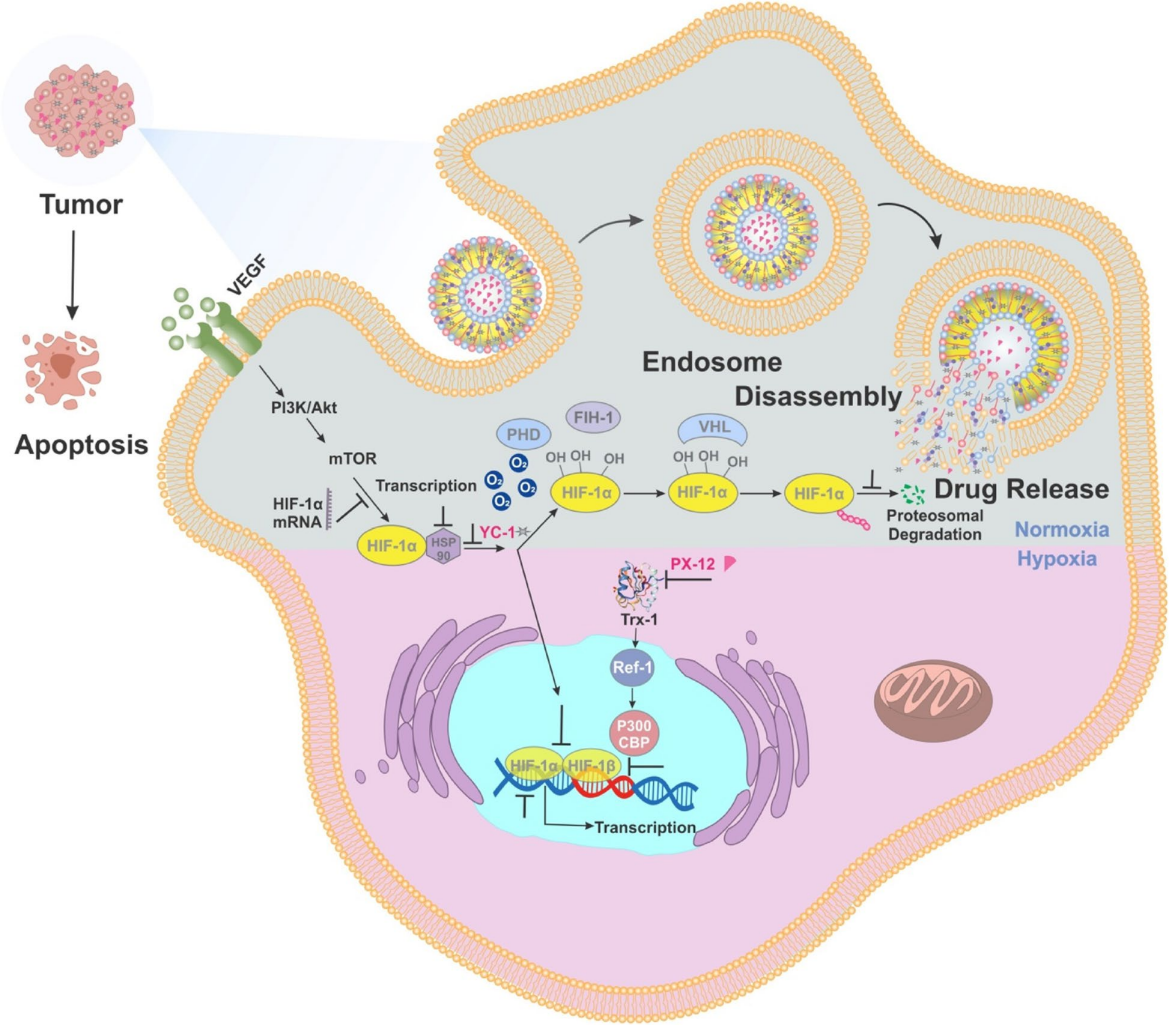

**Supplementary Information:**

The online version contains supplementary material available at 10.1186/s13036-023-00375-3.

## Introduction

Colon cancer, also known as colorectal cancer (CRC), is one of the most common cancers, affecting approximately 1.8 million people per year and the second leading cause of cancer-related deaths worldwide [1–3]. In addition to surgical removal of the tumor mass, both conventional (chemotherapy and radiotherapy) and innovative (personalized single-target therapies, immunotherapy, and photodynamic therapy (PDT)) approaches can be used to treat this type of cancer [4].

In human cells, O_2_ delivery and consumption are precisely regulated through the activity of hypoxia-inducible factors (HIFs) [5]. Increased levels of O_2_ consumption due to cell proliferation lead to hypoxia, which activates HIFs and results in the transcription of the VEGF gene, which encodes vascular endothelial growth factor to stimulate angiogenesis and thus increases O_2_ delivery. The characteristic features of cancer cells are dysregulated cell proliferation and structurally and functionally abnormal blood vessels, resulting in severe hypoxia [6]. To adapt to the hypoxic microenvironment, cancer cells must undergo physiological responses, including metabolic adaptation, increased resistance to apoptosis, and promotion of angiogenesis and metastasis, which together comprise the lethal cancer phenotype [7].

The main mechanism mediating adaptive responses to hypoxia is transcriptional regulation caused by hypoxia-inducible factor-1α (HIF-1α). The HIF protein is generally regulated in two ways: a) oxygen dependent and b) oxygen independent [8]. In oxygen-dependent regulation(normoxia), the presence or absence of oxygen is decisive [9]. Under normal conditions(in the presence of oxygen), HIF-1α is first hydroxylated by prolyl hydroxylase (PHD), then ubiquitinated by the VHL enzyme (a type of E3 ubiquitin ligase) and finally destroyed in the proteasome, so transcription of target genes does not occur. However, in hypoxia (In the absence of oxygen), the PHD enzyme is inactivated due to the lack of oxygen, so HIF-1α enters the nucleus, dimers with HIF-1β, and transcription is initiated [10, 11]. Non-oxygen-dependent pathways of HIF-1α regulation mainly regulate it at the transcriptional, post-transcriptional, or translational stage.PI3K/Akt/FRAP/mTOR,Trx-1,Topoisomerase I and II, HDAC,Hsp90, p53,Transition Metals, Nitric Oxide, Reactive Oxygen Species, are among the regulatory pathways independent of oxygen[9, 12, 13]. Because, most solid tumor cells undergo hypoxia and a number of cancer cell pathways are directed by hypoxia factor, HIF-1a targeted therapy may be a comprehensive approach against solid tumor cancers, in particular CRC.

YC-1 [3-(5ˊ-hydroxymethyl-2ˊ-furyl)-1-benzylindazole] is a synthetic molecule that was originally developed as an activator of guanylylcyclase (sGC) in platelets and revealed in vitro and in vivo antiplatelet activity through elevation of intracellular cGMP(Cyclic guanosine monophosphate) levels [14, 15]. Although YC-1 is required at higher concentrations for cGMP elevation, at lower concentrations, it inhibits HIF activity [16, 17]. Because of its inhibitory effect on HIF-1α, which is critical for tumor angiogenesis in a hypoxic microenvironment, YC-1 could be a potential candidate as a hypoxia-targeted agent [15]. The proposed mechanisms for the activity of YC-1in the inhibition of the HIF pathway include rapid degradation of HIF-1α [18] by inhibiting Mdm2 [19], which inhibits de novo synthesis of HIF-1α by inactivating the PI3K/Akt/mTOR pathway [20]. YC-1, as an inhibitor of the HIF-1α transcription factor, prevents the growth of many tumors such as breast cancer, prostate cancer, renal carcinoma, colon cancer and so on. According to studies that have already been done, YC-1 in a dose of 30–50 µM has significant toxicity which has a 50% survival rate [21].

Despite the large number of strategies developed against cancer, the self-protective mechanisms of tumors greatly limit the effectiveness of cancer treatment [22]. To overcome the obstacles, a promising antitumor chemical PX-12 (1-methylpropyl 2-imidazolyl disulfide) is used to inhibit the activity of thioredoxin 1 (Trx-1) by binding to cysteine ​​residue 73 of Trx [23, 24]. Trx-1 is an important protein that plays a critical role in maintaining cellular redox homeostasis and cell survival [25, 26]. It is regulated in a wide variety of cancers, such as colon and pancreatic cancers, leading to increased levels of Trx-1. Trx exerts its antioxidant effects through disulfide exchange [27]. In addition, PX-12 downregulates the expression of vascular endothelial growth factor (VEGF) by decreasing HIF-1α, thereby inhibiting cancer metastatic cells [28].

PX-12 is used as a therapeutic agent for advanced cancers which is now in phase II/IB clinical trials. IC50 values of PX-12 for cancer cells are on average between 6–30 µM [29]. The bioavailability for PX-12 is 1 according to Drug Bank database [30]. Therefore, it is potent to be used an orally bioavailable angiogenic inhibitor drug.

Considering the complex nature of cancer, the simultaneous administration of several drugs with different mechanisms of action can be an ideal strategy in cancer treatment compared to single-drug therapy. This combination therapy can synergistically inhibit tumor growth by modulating different signaling pathways and maximize the therapeutic efficacy of chemotherapy by minimizing the occurrence of multidrug resistance (MDR). In addition, it reduces the adverse side effects associated with high doses of single drugs [31, 32]. Despite the positive characteristics of combination therapy, this method shows low bioavailability and a lack of a tumor-specific strategy, which leads to reduced therapeutic efficacy and systemic toxicity [33]. Also, the inefficient co-delivery of multiple therapeutic agents to the tumor site due to differences in physiochemical properties and pharmacokinetic profiles is another possible reason that combination therapy lies in difficult [34].

The use of nano-sized drug delivery systems offers a new perspective for the effective encapsulation of multiple therapeutic agents with different physicochemical properties to achieve simultaneous delivery to the target site [31, 35]. The aforementioned delivery systems, named nanocarriers (NCs), can encapsulate hydrophobic as well as hydrophilic therapeutic agents, protecting them from degradation and facilitating their availability in the tumor site [36]. A wide variety of delivery systems have recently been developed that utilize traditional chemotherapeutic agents to improve their pharmacokinetic properties [37–41].

Niosomes, which are known as vesicular systems, are formed through the self-assembly of fatty acids and non-ionic surfactants in an aqueous solution with the help of physical stirring or high temperature [42]. The use of non-ionic surfactants in niosomes instead of phospholipids as membrane-forming components can overcome many disadvantages associated with liposomes, such as poor chemical stability, high production cost, susceptibility of phospholipids to oxidation, and the need for special storage and handling conditions [43]. Their specific structure, in which an inner aqueous core is surrounded by a hydrophobic bilayer membrane, allows the load and co-delivery of hydrophobic and hydrophilic drug molecules [44, 45]. Other striking strengths of niosomes are nontoxicity, non-immunogenicity, osmotically active, biocompatibility, and biodegradability [46]. Under physiological conditions, NPs such as noisome may enter the cells via passive and active transport. Passive transport of NPs into cells is relatively rare (eg, gold particles), and most NPs enter cells by endocytosis. Active uptake mechanisms in nonphagocytic cells including cancer cell lines are macropinocytosis, clathrin-mediated uptake, caveolae-dependent uptake and clathrin-independent caveolae-independent endocytosis. Endocytosis serves to absorb molecules from the extracellular space by invagination of the plasma membrane and formation of intracellular vesicles. Cellular uptake is influenced by size, shape, material and surface hydrophobicity besides surface charge. Studies on the effect of charge density and of the kind of charge (positive, negative) in nonphagocytic cells such as HT-29 cancer cell showed that some charged nanoparticles are taken up better than their uncharged counterparts [47]. Considering the framework of criteria mentioned above, the authors, for the first time, tried to prepareYC-1 (hydrophobic drug) and PX-12 (hydrophilic drug)-loaded noisome as an effective chemotherapeutic agent to inhibit the HIF pathway. In this study, the niosomal formulations of YC-1 and PX-12 were developed and optimized by the central composition method (CCD) using the Box-Behnken design. The synthesized niosomes was characterized in terms of physicochemical properties. The stability of the dual drug-loaded niosomes was also studied in two storage conditions for two months. The release profile and release kinetics of the NIO/PX-YC were examined. Then the cytotoxicity of the resulted nanocarrier on HT-29 colon cancer cells and HFF cells (as control normal cells) was evaluated. In addition, the induction of apoptosis and inhibition of cell growth on HT-29 cells was assessed. Finally, HIF-1α gene expression and cellular protein levels of HIF-1α in HT-29 cells were evaluated by q RT-PCR and western blot.

## Material and methods

### Material

Chloroform, ethanol, methanol, span 60, Tween 60, cholesterol, dimethyl sulfoxide (DMSO), ultrapure sodium dodecyl sulfate (SDS), and Amicon® Ultra Centrifugal Filter Devices (Amicon Ultra-15 Membrane MWCO 30 KDa) were purchased from Merck company (Germany). Phosphate buffered saline tablet (PBS) provided by Bio Basic Company(Canada). The PX-12 (PubChem CID: 219,104), YC-1(PubChem CID: 5712), DEPC water, and dialysis membrane (MWCO 12 KDa) were obtained from Sigma Aldrich company(USA). Dulbecco's Modified Eagle's medium (DMEM), Trypsin–EDTA, and Penicillin–Streptomycin(pen-strep) were received fromBiosera company (France). Fetal bovine serum (FBS) was bought from Gibcocompany (USA)0.3-(4,5-dimethylthiazol-2-yl)-2,5-diphenyltetrazolium bromide (MTT) was purchased from Atocelcompany(Australia).ApoFlowEx FITC Kit (Annexin V/PI) was acquired from EXBIO company (Czech Republic). The flow cytometer used in this study is a FACScalibur belonging to BD Biosciencescompany (Canada). Sybr Green master mix real-time PCR was purchased from Ampliqoncompany (Denmark). Intestinal cancer cells (HT-29) and human foreskin fibroblast (HFF) were supplied from the National Center for Genetic and Biological Resources of Iran.

### Optimization of noisome formulation

The central composition method (CCD) was used to optimize the niosomal formulations through the Box-Behnken design. To investigate the relationship between a set of independent variables and dependent variables by fitting the data using a polynomial equation, two numerical parameters lipid content (total surfactant and cholesterol) µmol and surfactant to cholesterol molar ratio, were selected for the study.

The effect of their concentration on niosomal particle size (nm), polydispersity index (PDI), entrapment efficiency percentage (EE%), and drug release percentage was investigated. The polynomial equation was obtained using Design-Expert software (Version 7.0.10, Stat-Ease, Inc., Minneapolis, MN, USA). Comparisons were made between the experimental data and the predicted responses. Using the point prediction method, the optimal formulation was chosen for further study. Table 1 represents these factors and their levels.

**Table 1:** Different levels for variables in the CCD optimization^a^

Level	-1	0	+ 1
A (Lipid content, µmol)	200	250	300
B (Surfactant: Cholesterol, molar ratio)	0.5	1	2

^a^Sonication time:5 min; Span60:Tween60 = 1:1

### Preparation of niosomal formulations

The thin-layer hydration method was used for the synthesis of different formulations of niosomes containing PX-12 and YC-1. Due to the hydrophobic property of YC-1, it is added in the first step along with other nisome components. Briefly: YC-1, Cholesterol, span 60 and tween 60 are dissolved in 10 ml of organic solvent (chloroform /methanol with a ratio of 2:1). The solution was transferred to a 50 ml round bottom flask. The organic solvent was evaporated under vacuum using a rotary evaporator (Heidolph Instruments, Schwalbach, Germany) at 60°C and 150rpm for 30min until a thin dried film was formed in the bottom of the flask. Afterward, because PX-12 is hydrophilic, it is added to the solution in the last steps along with PBS. PBS containing PX-12(10 ml, PH 7.4) was used to hydrate dry, thin films, and the mixture was stirred at 60 °C and 150 rpm for 30 min. (Both drugs have concentration of 50 µg/ml). Finally, the sample was sonicated for 5 min (Hielscher UP50H ultrasonic processor, Germany, Amplitude: 25%, 200 w) to obtain the niosomal formulations with uniform size distribution. The samples were stored in a refrigerator (4°C) for future use.

### Physicochemical characterization of niosomal formulations

Assessment of some critical parameters of formulations such as the particle size distribution, the polydispersity index (PDI), and zeta potential was performed by Dynamic Light Scattering technique (DLS) (Zetasizer Nano S90, Malvern Panalytical Ltd., Malvern, United Kingdom). All of these parameters were investigated in a nano-zeta sizer device equipped with a green laser with a wavelength of 633 nm at a temperature of 25 °C. Surface features and morphology of niosomes are evaluated by Field Emission Scanning Electron Microscopy (FESEM) and Transmission Electron Microscopy (TEM). For examination by FESEM microscope (SSX-500, Shimadzu, Japan), about 1 ml of niosomal formulation solution was dried by freeze dryer. For TEM, a drop of 0.1% w/v niosomal solution was placed on a carbon-coated copper grid and stained with a 1% phosphotungstic acid. The stained niosomes were imaged with TEM (Model EM900, Zeiss Microscopy, Jena Germany). To determine the chemical composition of niosomal formulations and the possible interactions between the carrier and drugs Fourier Transform Infrared Spectrometer (FTIR) (Spectrum Two, USA) was used. Lyophilized samples were mixed individually in KBr, and the pellets were formed by placing the samples in a hydraulic press. FTIR analyses were accomplished in the scanning range of 4000 to 400 cm^−1^ at room temperature. UV–Visible absorption spectra were obtained on a UV–Visible spectrophotometer (UV-1700 PharmaSpec, Shimadzu, Kyoto, Japan).

### Entrapment Efficacy (%EE)

The ultrafiltration method has been used to investigate entrapment efficiency. For this, niosomal formulations of drugs were filtered at 4°C and 4000rpm for 20 min in a centrifugal filter tube (Amicon Ultra-15-Membrane MWCO 30000 DA). The free drug passes through the filter pores, and what remains on the filter is the niosome containing the drugs. The absorbance of the sample passing through the filter is read at 250 and 270 nm for PX-12 and YC-1, respectively, using a UV–Visible spectrophotometer. The percentage of encapsulation efficiency is calculated according to Eq. 1:

1 $$\mathrm{EE\%}=\frac{\mathrm{The\;amount\;of\;the\;initial\;drug}-\mathrm{The\;amount\;of\;free\;drug}}{\mathrm{The\;amount\;of\;the\;initial\;drug}}\;\mathrm{x}\;100$$

### In vitro drug release study

In vitro release profile of PX-12 and YC-1 from niosomal formulation (NIO/PX-YC) was conducted using a semipermeable acetate cellulose dialysis bag (MWCO 12kDa) in phosphate buffer saline containing 0.5% w/v SDS. Briefly, two dialysis bags containing a fixed amount of drug-loaded niosomal solution were placed in 25 ml of PBS-SDS solution with pH 5.4 and 7.4 and incubated at 37°C with a gentle shaking of 50 rpm. At predetermined time intervals (1, 2, 4, 6, 8, 24, 48, and 72h), 1 ml of the release medium was withdrawn and replenished with the same volume of fresh PBS-SDS. Finally, the amount of PX-12 and YC-1 released from the niosomal formulations was determined by UV–Visible spectrophotometer at 250 and 270 nm, respectively and the cumulative release of drugs was calculated. For the comparative study, the experiment was repeated for the free drugs (PX-12, YC-1) as a control with the same concentration of their niosomal formulation. The experiments for each group were conducted three times and the results were represented as mean ± SD. The amount of cumulative release percentage (CR) was calculated according to the following equation (Eq. 2, 3) [48]:

2 $$CR \left(\%\right)= \frac{Vs}{V} {P}_{t-1}+ {P}_{t}$$

3 $${P}_{t}= \frac{{C}_{i}V}{M} \times 100$$

where $Vs$ is the volume of sample withdrawn (ml), $V$ is the bath volume (ml), ${C}_{i}$ is drug concentration at time t (µg/ml), $M$ is the total amount of drug (µg), ${P}_{t}$ and ${P}_{t-1}$ represents drug release percentage at times t and t-1. The experiment was repeated using free drugs as a control with the same concentration of their niosomal formulation.

### Release kinetics study

To describe the release kinetics of PX-12 and YC-1 from noisome various mathematical models were used. Zero-order(Eq. 4), where the drug release rate does not depend on its concentration [49]; the first order (Eq. 5), where the drug release rate is concentration-dependent [50]. Korsmeyer-Peppas (Eq. 6) describes the drug release from a polymeric system [51]. Higuchi (Eq. 7) describes the release of drugs from the insoluble matrix as a square root of a time-dependent process based on the Fickian diffusion [52].

4 $$C={k}_{0}t+{C}_{0}$$

where C is drug concentration at time t, C_0_ is the initial drug concentration, t is the time, and k_0_ is the zero-order rate constant expressed in units of concentration/time.

5 $$\mathrm{ln}C-\mathrm{ln}{C}_{0}= {k}_{1 }t/2.303$$

where k_1_ is the first-order rate constant.

6 $$\frac{{M}_{t}}{{M}_{\infty }}= {K}_{kp}{t}^{n}$$

where Mt/M∞ is the fraction of drug released at time t, K_KP_ is the rate constant, and n is the release exponent.

7 $$C={K}_{H }\sqrt{t}$$

where constant K_H_ reflects system design variables.

### Stability

The NIO/PX-YC was stored under two different storage conditions room temperature (25 ± 2 °C) and refrigerator temperatures (4 ± 2 °C) for two months to evaluate its stability. During the storage time, their physicochemical properties, including particle size, PDI, and EE%, were analyzed at certain time intervals (0, 30, and 60 days).

### MTT assay

HT-29 and HFF cells were cultured in DMEM/F12 medium which was supplemented by adding 10% fetal bovine serum (FBS), 100 U/ml penicillin and 100 μg/ml streptomycin in an incubator having humidified atmosphere. The gas content and tempratue of incubator were 5% CO_2_ and 37°C respectively.

The cells were seeded into 96-well plates at a density of about 10^4^ cells per well and incubated for 24 h. Subsequently, the culture medium was replaced by the fresh medium containing different concentrations of drug formulations (free PX-12, free YC-1, free drugs combination, NIO/PX-YC). YC-1 dissolved in cell culture safe range of DMSO (< 0.1%) due to its hydrophobic property, and PX-12 dissolved in medium because of its hydrophilicity and enough water solubility. To assess the biocompatibility of the niosomes, the cells were treated with non-loaded niosome. The untreated cells with culture medium were used as a control group. After 24, 48, and 72 h incubation, the culture medium was aspirated and replaced with MTT solution (5 mg/ml, 20 µl) and was incubated for 4 h. Incubation for HFF cells was performed for 48 and 72 h. Following incubation, the medium was removed, and 100 µl DMSO was added to dissolve the obtained formazan crystals. Finally, absorbance was measured at 540 nm using an ELISA plate reader (Multiskan MK3, Thermo Electron Corporation, USA). The viability percentage was calculated according to Eq. 8:

8 $$\mathrm{Cell\;Viability }(\mathrm{\%})=\frac{\mathrm{ absorbance\;of\;treated\;cells }}{\mathrm{ absorbance\;of\;control }}\mathrm{x}100$$

### Apoptosis assay

To evaluate the apoptosis/necrosis ratio, Annexin V-FITC dual staining method was used. First, the IC_50_concentration of free drugs and drug formulations was used to treat the cells for 24 and 48 h, then Annexin V/Propidium Iodide (PI) kit was used to differentiate apoptotic and normal cells according to the manufacturer's instructions. The cells were washed twice with cold PBS, and the pellet was re-suspended in 100 µl of 1 × Annexin binding buffer (5 × 10 ^5^ cells/well). Next, 5μl of Annexin V- FITC and PI was added to each sample. After 15 min of incubation (25°C), the cell suspension was centrifuged, and the resulting pellet was re-suspended in 100 µL of 1 × Annexin V Binding Buffer. The cells without any treatment were considered a control group. Finally, the levels of apoptotic/necrotic cells were evaluated using flow cytometry. The samples were analyzed in three replications using the BD FACSDiva instrument and Flow Jo software (BD Biosciences company, Canada).

### Cell cycle analysis

The proliferation of cells and the cell cycle process were evaluated by propidium iodide (PI) staining. Cells were seeded in the complete medium in 6-well plates at a density of about 1 × 10^6^ cells/well and incubated for 24 h. The cells were then treated with the IC_50_ concentration of free drugs and drug formulations for 24 and 48 h. Following incubation, the cells were fixed with cold ethanol 70% and stored overnight at 4°C. Then, they were collected by centrifuging (300rpm, 5 min), washed with PBS, and stained with 1 ml of PI master mix (containing 40μl PI, 10μlRnase, 950μlPBS) in the dark. The cells were then incubated for 30 min at room temperature and evaluated by flow cytometry. The data were analyzed by FlowJo software.

### Gene expression analysis by q RT-PCR

The cells (1 × 10^8^ cells/well in a 6-well plate) were treated with IC_50_ concentration of free drugs and drug formulations for 72 h. Un treated cells were considered as a control group. Trizol reagent was used to extract total RNA according to the manufacturer's procedures and quantified with gel electrophoresis and nanodrop (Thermo Scientific, Waltham, MA, USA). Revert Aid First Strand cDNA transcription Kit (Fermentas, Vilnius, Lithuania) was used to transcript extracted total RNA to cDNA. The mixture contained reaction buffer (2µl, 2X), total RNA (5 µg), reverse transcriptase enzyme (1 µl), deoxynucleotide triphosphate mixture (1µl), random hexamer (1 µl), DEPC water (up to the final volume of 20 µl). The mixture was incubated at 25 °C (10 min), 47 °C (60 min), and 85 °C (5 min). subsequently, it was kept on ice until use. The primers sequence for target genes (HIF-1α and β-actin) is presented in Table 2. β-actin gene was used as an internal control. Finally, qRT-PCR was done by SYBR Green Master Mix (Applied Ampliqon, Danmark) using Bioneer Real-Time PCR equipment (Korea). The cDNAs, primer, and master mix cocktail were incubated according to the following temperature program: 95 °C for 3 min, followed by 40 cycles at 95 °C for 10 s and 20 s at 60 °C and 72°C at 20 secs. The results were reported as fold changes that were calculated by the ΔΔCт method.

**Table 2:** The sequence of primers used in PCR

Genes	Accession	Forward primer (5′–3′)	Reverse primer (3′–5′)
HIF1-α	NM_001530	CCACTGCCACCACTGATGAA	CTGCTCTGTTTGGTGAGGCT
β-Actin	NM_001101.5	TCCTCCTGAGCGCAAGTAC	CCTGCTTGCTGATCCACATCT

### Western blot analysis

Western blot analysis was performed to investigate the HIF-1α protein in HT-29 cell line in hypoxia conditions. For this purpose, 2 × 10^5^cells were seeded in each well of 6-well plates. After 24 h, the cells were treated with IC_50_ concentrations, similar to the previous tests. The plate was transferred to the hypoxia incubator (1% O_2_, 5% CO_2_, 94% N_2_) for 24 h. Following incubation, the cells were harvested, washed with ice-cold PBS, and then scraped and centrifuged at 1500 rpm for 5 min. The harvested cells were lysed using RIPA buffer having the following reagents: Tris–HCL, SDS, Protease inhibitor cocktail EDTA, sodium deoxycholate, NaCl, and 1% Triton X-100 or NP40, then centrifuged (10,000 rpm, 10 min). The proteins in the supernatant were collected and stored at -20°C. After quantification by Bradford assay, round 10 µg of proteins were separated by SDS-PAGE and subsequently transferred to nitrocellulose membrane overnight. Blocking of the membranes was performed with 0.5% Tween-PBS and 2% low-fat dried milk. The blocked membranes were then incubated with primary antibodies against β-actin (Santa Cruz Biotechnology Inc. Dallas, USA) or HIF-1α (Santa Cruz Biotechnology Inc. Dallas, USA) for 16–18 h at 4°C followed by washing and incubation with secondary antibodies (Santa Cruz Biotechnology) mouse anti-rabbit IgG-HRP at a dilution of 1:10,000 (Santa Cruz Biotechnology, Inc, Dallas, USA), for 2h. Finally, a chemiluminescence reagent, ECL (Elabscience company, USA), was added, and the bands emerged in the dark room detection system. After the films appear, the appeared protein bands are analyzed with Image J software (USA).

### Statistical analysis

Data were reported as mean ± SD, and the graphs were plotted using GraphPad Prism version 8 and Design-Expert software (Version 7.0.10.). Data were statistically analyzed using analysis of variances (ANOVA), and a p-value less than 0.05 was considered a significant difference.

## Result

### Optimization of noisome formulation

**Fig. 1:**
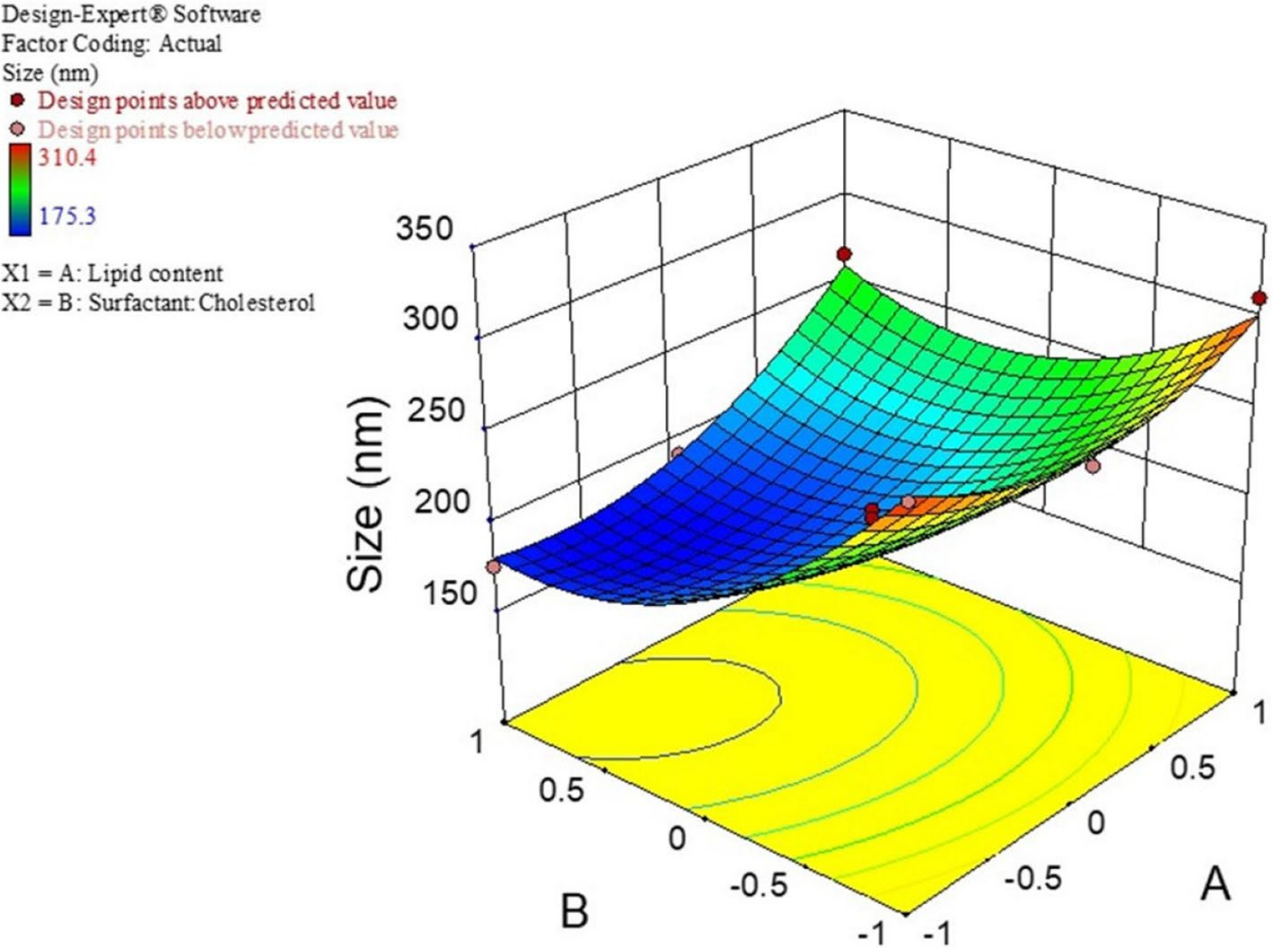
CCD method for average diameter as a function of lipid content and surfactant to cholesterol molar ratio

**Fig. 2:**
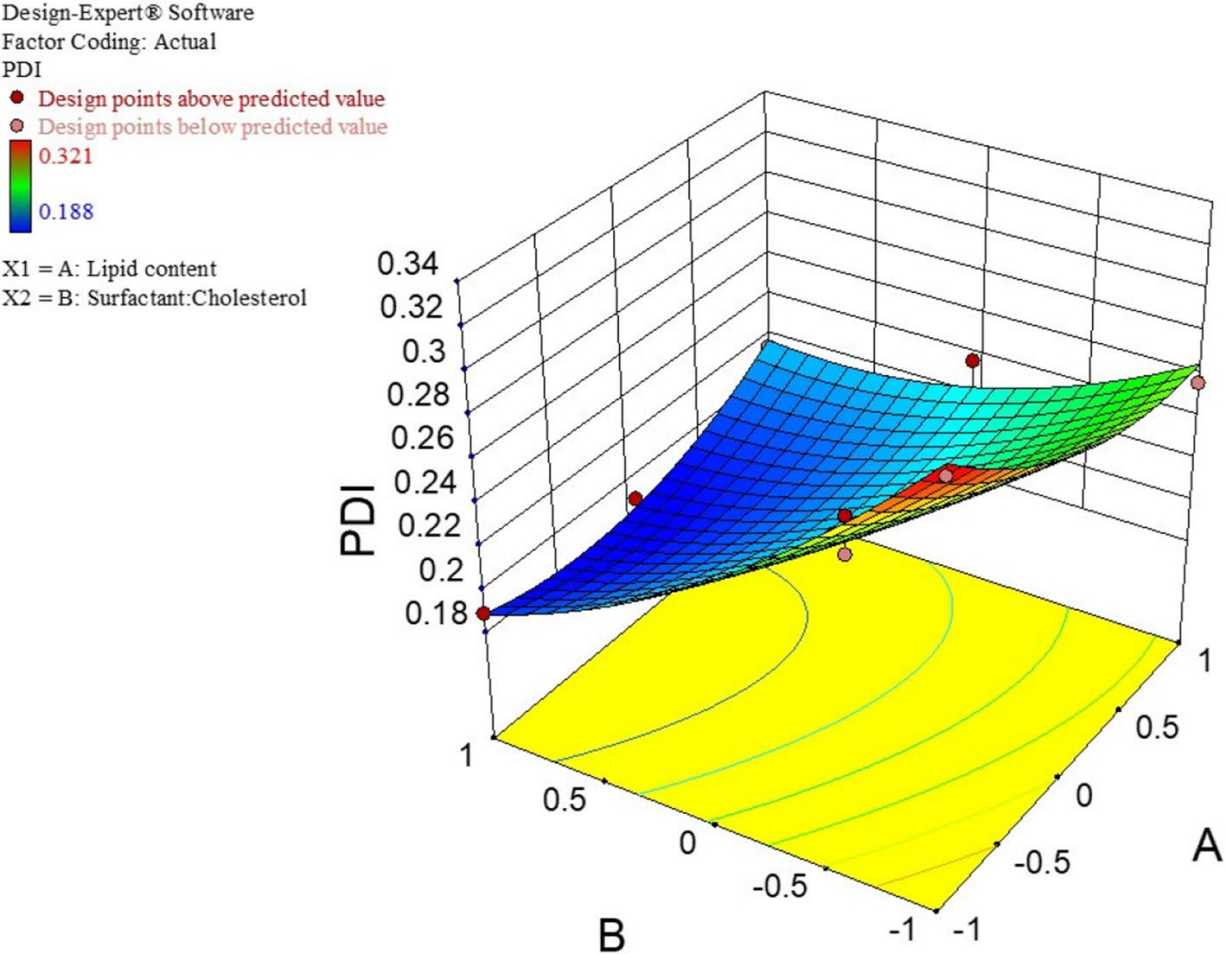
CCD method for PDI as a function of lipid content and surfactant to cholesterol molar ratio

The mean particle size, PDI, and %EE for all 11 experiments were fitted to the quadratic model. In these experiments for the optimization process, the lipid content(A) and surfactant/cholesterol molar ratio (B) were considered independent variables. In contrast, The EE%, particle dimension, and PDI were used as dependent variables. Table 3 represents the results of optimization tests. The size and PDI of NIO/PX-YC are in the range of 175.3 to 310.4 nm and 0.188 to 0.321, respectively. Also, the EE% in dual drug formulation for PX and YC are from 71.29 to 93.68 and 50.35 to 75.41, respectively. The analysis of variance for particle size is depicted in Table S1. The response is a quadratic model and is considered significant (*p* < 0.05). The independent variables A (lipid content) and B (surfactant to cholesterol molar ratio) significantly affected the particle size. The summary of results for regression analysis, along with the regression equation, is shown in Table S2. Also, Fig. 1 demonstrated the response surface plot of particle size for NIO/PX-YC nanoparticles. It can be concluded that by decreasing the lipid content and surfactant to cholesterol molar ratio or increasing both the particle size increases. Statistical analysis and regression analysis of PDI is represented in Table S3 and S4. Similarly, the response for the quadratic model is significant (*p* < 0.05), and also PDI is significantly affected by independent factor B. According to the response surface plot of PDI for NIO/PX-YC nanoparticles with increasing molar ratio of surfactant to cholesterol, the PDI of nanoparticles decreases (Fig. 2). Data obtained from statistical analysis and regression analysis of EE% for PX-12 and YC-1 in dual drug formulation are depicted in.

**Table 3:** CCD-Behnken method to design experiment for NIO/PX-YC

Run	Levels of independent variables		Dependent variables			
	A	B	Average size (nm)	PDI	EE_PX_ (%)	EE_YC_ (%)
1	1	0	226.2	0.237	91.54	57.49
2	-1	-1	299.5	0.321	71.29	50.35
3	0	-1	267.1	0.292	87.39	57.24
4	-1	1	175.3	0.189	84.12	69.55
5	1	1	265.3	0.214	93.68	75.41
6	1	-1	310.4	0.257	85.21	60.92
7	0	1	190.1	0.188	90.29	74.91
8	0	0	195.4	0.214	85.21	64.25
9	-1	0	211.1	0.245	80.54	62.25
10	0	0	200.3	0.212	87.36	67.31
11	0	0	188.6	0.195	88.42	66.19

**Fig. 3:**
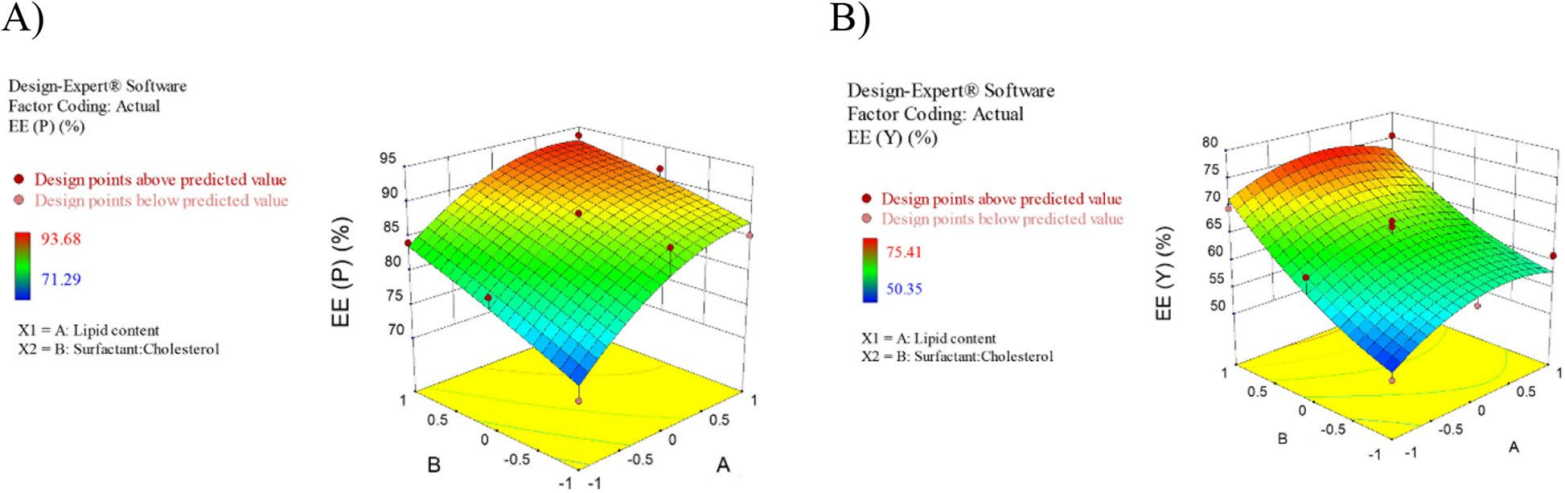
CCD method for **A**) EE_PX-12_, **B**) EE_YC-1_ as a function of lipid content and surfactant to cholesterol molar ratio

Table S5 and S6. Like the other dependent parameters, the response for the quadratic model is significant (*p* < 0.05) for both drugs. As can be seen, EE% is widely varied by the independent factor A and B for PX-12 and B for YC-1. Interestingly, in all independent variables, the F value of the model, which refers to the quadratic pattern, is significant. As shown in Fig. 3, increasing the lipid content and molar ratio of surfactant to cholesterol led to an increase in EE percentage for PX-12. However, for YC-1, increasing the molar ratio of surfactant to cholesterol significantly increases the amount of EE.

By applying the limits of responses, the optimal formulation with a good desirability index was obtained (Table 4). The predicted values for the particle size, PDI, and EE% (PX-12, YC-1) in the optimized formulation were 197.372 nm, 0.188 mv, 92.141 for PX-12, and 75.41 for YC-1, respectively, which were very close to the experimentally observed values (Table 5).

**Table 4:** Desirability criteria and predicted values for the variables

Number	A(lipid content, mg)	B(surfactant/cholesterol molar ratio)	Desirability
1	259.2	1.961	0.920

**Table 5:** The optimized responses obtained by the CCD method and the experimental data for the same responses under the optimum conditions

Parameter	Predicted by Box-Behnken	Experimental Data
Average size (nm)	197.372	185.00 ± 5.40
PDI	0.188	0.179 ± 0.012
Entrapment Efficiency (EE_PX_) (%)	92.141	91.24 ± 1.15
Entrapment Efficiency (EE_YC_) (%)	75.410	78.36 ± 1.54

### Preparation of NIO/PX-YC nanocarrier

**Fig. 4:**
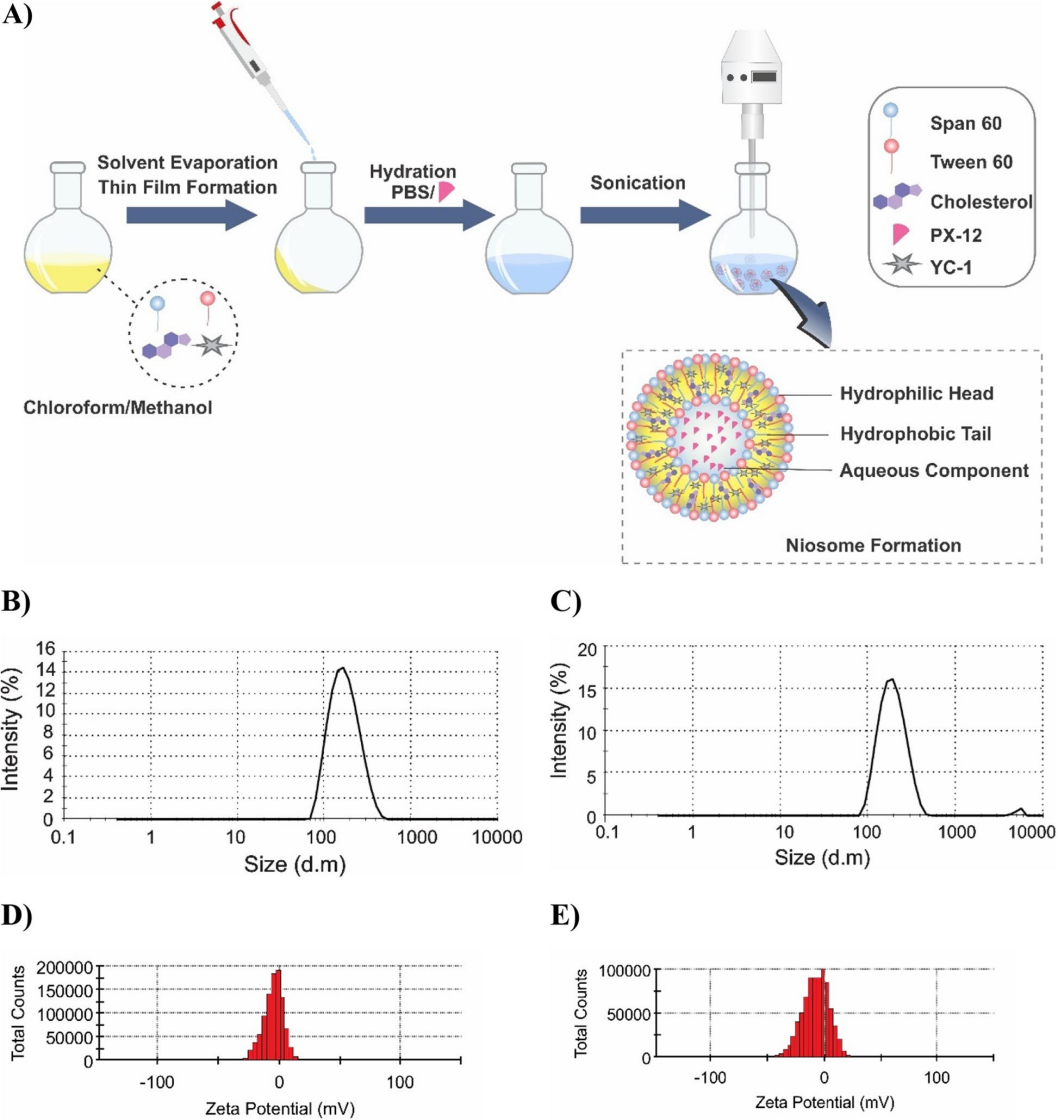
**A** Schematic illustration for the preparation of optimized niosomes by thin-layer hydration method. **B-C** Hydrodynamic size of blank noisomeandNIO/PX-YC, respectively. **D-E** Zeta potential for blank noisome and NIO/PX-YC, respectively

In this work, the optimal niosomal formulation of cholesterol, span 60, and tween 60 containing PX-12 and YC-1 were prepared through a thin-layer hydration method. The synthesis route is depicted in Fig. 4A.

### Size distribution, PDI and zeta potential of the niosomes

Average hydrodynamic particle size, PDI, and zeta potential were measured by DLS. The average particle size of the blank niosome was found to be 158.5 nm. The entrapment of drugs into the niosome (NIO/YC-PX) resulted in an increase in the size of the niosome to 185 nm. The PDI for blank noisome and NIO/YC-PX was 0.137 and 0.179, respectively. Also, the zeta potential of the blank niosome was observed to be -4.56 mV, while those for the NIO/YC-PX were observed to be -7.10 mV (Fig. 4B-E).

### FTIR analysis

**Fig. 5:**
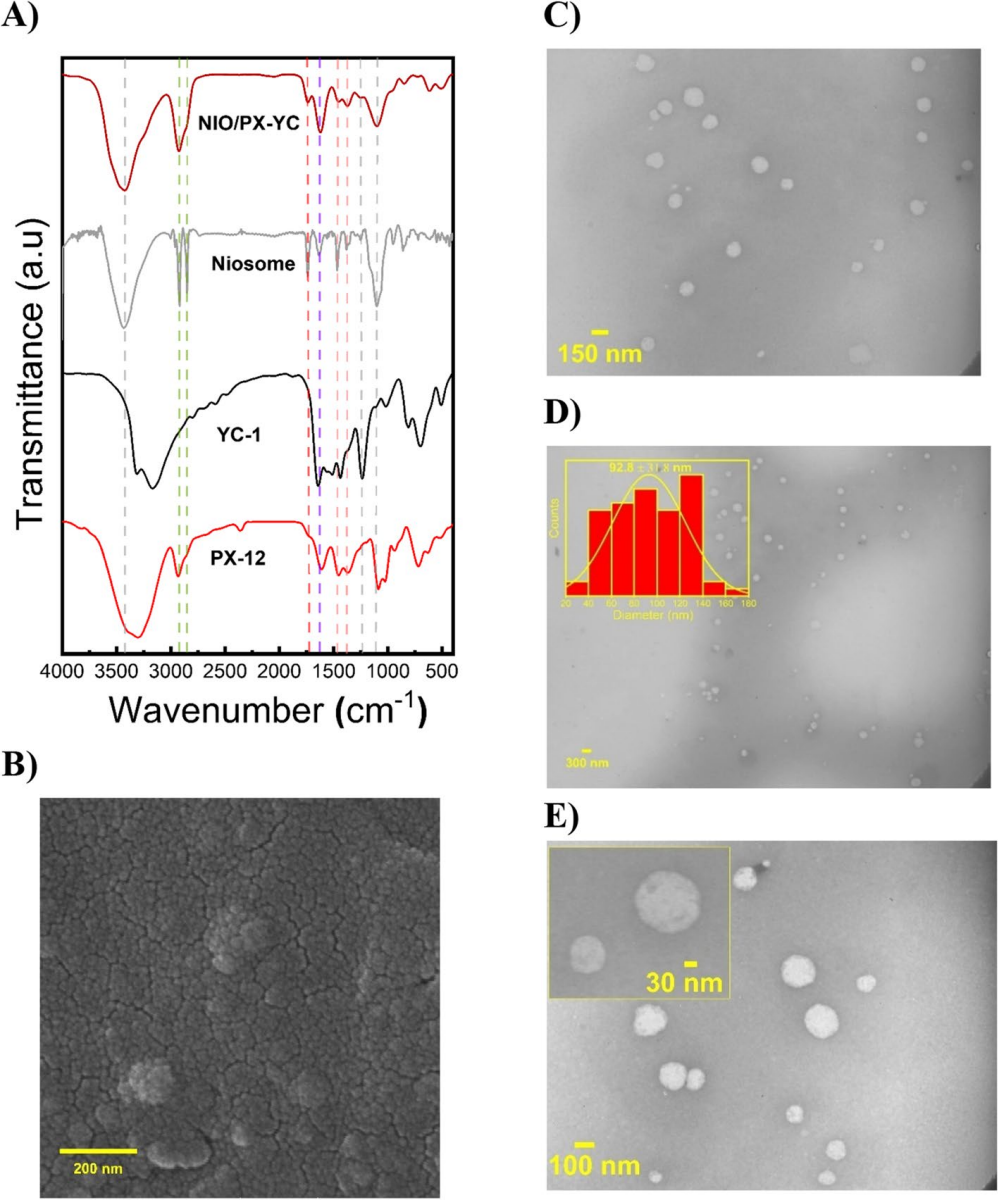
**A** FTIR spectra of NIO/PX-YC, blank noisome, YC-1, and PX-12. **B** FESEM micrograph of the niosomes. **C-E** TEM images representing the structure of niosomes with a corresponding size distribution histogram

The FTIR spectra of the NIO/PX-YC, blank noisome, YC-1, and PX-12 are presented in Fig. 5A. As depicted in the figure, the spectrum of the blank noisome has characteristics peaks of its component cholesterol, tween 60, and span 60, that include -OH stretching vibration at 3438 cm^−1^, aliphatic C-H stretching bond at 2850 and 2919 cm^−1^, tween 60 and span 60 -C = O stretching bond at 1737 cm^−1^, C = C stretching vibration at 1637 cm^−1^, C-H bending mode at 1467 and 1380 cm^−1^, and C-O stretching vibration at 1253 and 1101 cm^−1^. After loading the tumor inhibitor compounds into the noisome, the appearance of the spectrum does not show any observable changes. However, the intensity of these characteristic peaks changed. In addition, the peaks of YC-1 and PX-12 are not easily observed in the spectrum because they are masked with the broad peaks of characteristic niosome. Altogether, these indicate preferential hydrophobic/hydrophilic interactions of YC-1 and PX-12 with the niosome that confirms the successful loading of these compounds.

### Morphological studies of the niosomes

The morphological appearance of drug-loaded niosomes was assessed by FESEM and TEM. As depicted in Fig. 5B-E niosomes have a uniform spherical shape with a smooth surface and separated firm boundaries. The images obtained from the TEM microscope also confirm the spherical nature of the niosomes. Based on the TEM images, the average diameter of the particles is 92.8 ± 31.8 nm.

### Entrapment efficiency

In this study, we used the thin-layer hydration method to incorporate PX-12 and YC-1 into the optimized niosomal formulation. The entrapment of drugs into the niosome happens through electrostatic and hydrophobic interactions. The entrapment efficiency for YC-1 and PX-12 encapsulation in the NIO/PX-YC were found to be 78.36 ± 1.54 and 91.24 ± 1.15, respectively (Table 5).

### In vitro drug release and kinetic modeling studies

**Fig. 6:**
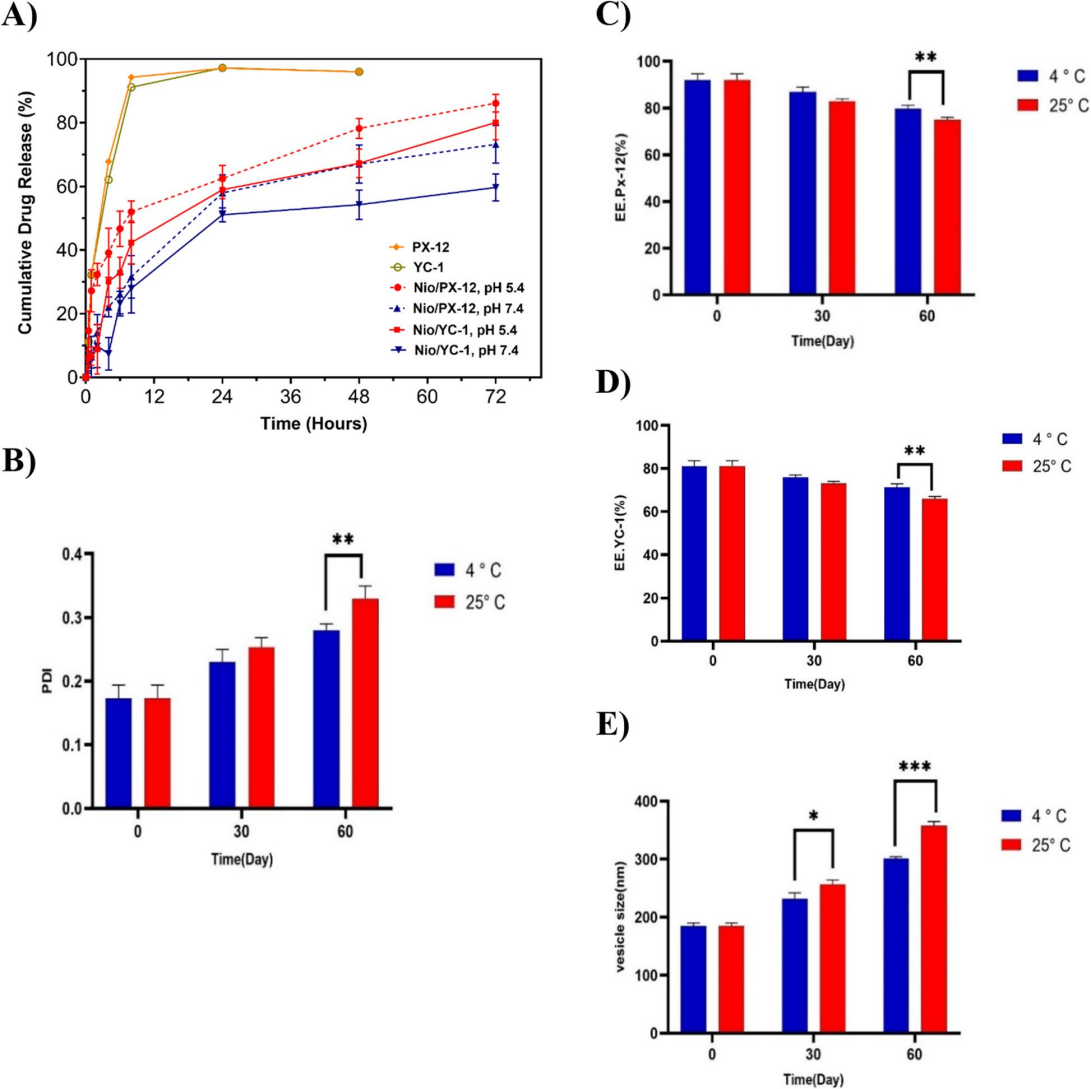
**A** In vitro release profile of PX-12 and YC-1 from the NIO/PX-YC nanocarriers along with their comparison with free drugs in pH 7.4 and 5.4 at 37 °C. **B-E** Stability of optimal NIO/PX-YC formulation stored during 1 and 2 months at 4 ± 2 °C and 25 ± 2 °C. The data are shown based on (mean ± SD). *p < 0.05, **p < 0.01, and ***p < 0.001

The release profile of PX-12 and YC-1 from niosomes was studied in PBS-SDS medium (as release medium) under physiological and acidic conditions for 72 h. As illustrated in Fig. 6A, free PX-12, and YC-1 had a burst release of 94.25% and 91.12%, respectively, after 8 h of incubating at 37 °C; and reached the monotonous rate after 24 h. Also, the release diagram of NIO/PX-YC showed a biphasic release profile for both drugs. The initial burst release during 8 h as a result of the drug’s diffusion from the outer layer of the niosomes, followed by a steady release of drugs within 72 h. Interestingly, compared to the release percentage of free drugs after 8 h, the encapsulation of PX-12 and YC-1 into the noisome decreased the release percentage to 34.62% and 27.92%, respectively, at pH 7.4. In contrast, the release percentage for the niosomal formulation of drugs at pH 5.4 increased to 51.98% and 42.35%, respectively during the same time of incubation. After 72 h, the release percentage at pH 5.4 reached 86.14% and 80.09%, respectively, which could be attributed to the breaking of niosomes as a result of swelling in acidic conditions [53]. In the acidic tumor microenvironment, drug-loaded niosomes are disrupted, and the rate of release begins to increase, resulting in increased tumor toxicity and relative safety for physiological pH tissue.

The release kinetics of PX-12 and YC-1 in aqueous form and niosomal formulation are listed in Table S7. The free drugs follow the first-order kinetic models, in which the drug release rate depends on its concentration. According to the results, Korsmeyer–Peppas model could reliably estimate the release behavior of PX-12 and YC-1 from NIO/PX-YCnanocarriers in both physiological and cancerous conditions. The release exponent of drugs in pH 7.4 and 5.4 indicate the anomalous transport mechanism of drugs from the niosomal formulation, which refers to the combination of Fickian diffusion and erosion [54].

### Stability studies

To examine the physical stability of the optimal NIO/PX-YCformulation EE%, PDI and vesicle size were studied at days 0, 30, and 60 in two storage temperatures 4 and 25 °C. According to Fig. 6B-E, by increasing the storage time, an increase in particle size and PDI, along with a decrease in EE% (for both drugs), is observed. But the stability of the sample stored at 4 ± 2 °C is greater than the sample stored at 25 ± 2 °C, which could be ascribed to the higher stability of the hydrophobic niosomes at lower temperatures [55].

### In vitro cytotoxicity studies

**Fig. 7:**
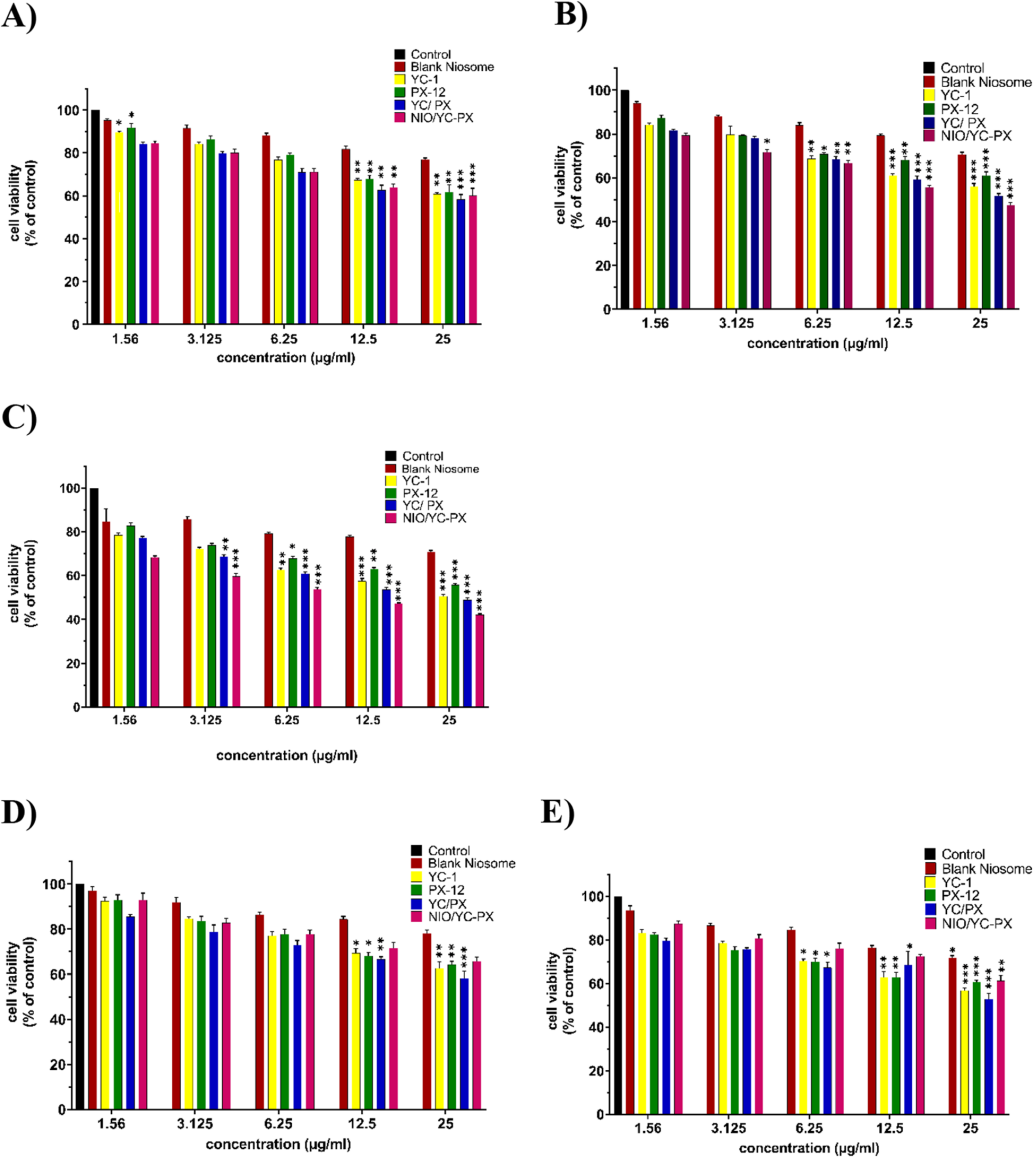
**A-C** Cell viability of HT-29 cells being exposed to different concentrations of PX-12, YC-1, PX/YC, NIO/YC-PX, and blank niosome after 24 h, 48 h, and 72 h, respectively. **D-E** Cell viability of HFF cells being exposed to different concentrations of PX-12, YC-1, YC/PX, NIO/YC-PX, and blank noisome after 24 h, 48 h, and 72 h, respectively. All tests were repeated three times, and the data are shown based on (mean ± SD). *p < 0.05, **p < 0.01, and ***p < 0.001

In vitro cytotoxicity of HT-29 and HFF cell lines was investigated after being treated with different formulations of drugs for 24, 48, and 72 h. As shown in Fig. 7, no obvious evidence of toxicity was observed for the niosomal platform without loaded drugs in comparison to the control group, indicating the cytocompatibility of the nanocarrier. On the other hand, the viability of the HT-29 cells treated with different concentrations of free drugs and their combination, along with their niosomal formulation, shows a dose-dependent and time-dependent effect (Fig. 7A-C). Combination treatment of cells resulted in a significant loss in cell viability. More interestingly, the enhanced cytotoxicity of the cells treated with NIO/YC-PX in comparison to free drugs and their combination indicates the ability of nanocarrier to increase drug entrance inside the cancer cells through the endocytosis process, as a result, decrease the appropriate drug dosage and adverse systemic toxicity. In the HFF cell line, the NIO/YC-PX did not induce any significant toxicity in normal HFF cells following a 72 h treatment (Fig. 7D, E). The IC_50_ values of all formulations are depicted in Table 6.

**Table 6:** The IC_50_ value of different drug formulations in HT-29 cells at 48 and 72 h

Time (hours)	IC_50_(µg/ml)				
	YC-1	PX-12	YC/PX	NIO/YC-PX	Blank niosome
48	25	52.134	24.36	75.13	934.65
72	23.78	27.54	22.65	16.32	789.64

#### Flow cytometric analysis of cell death

**Fig. 8:**
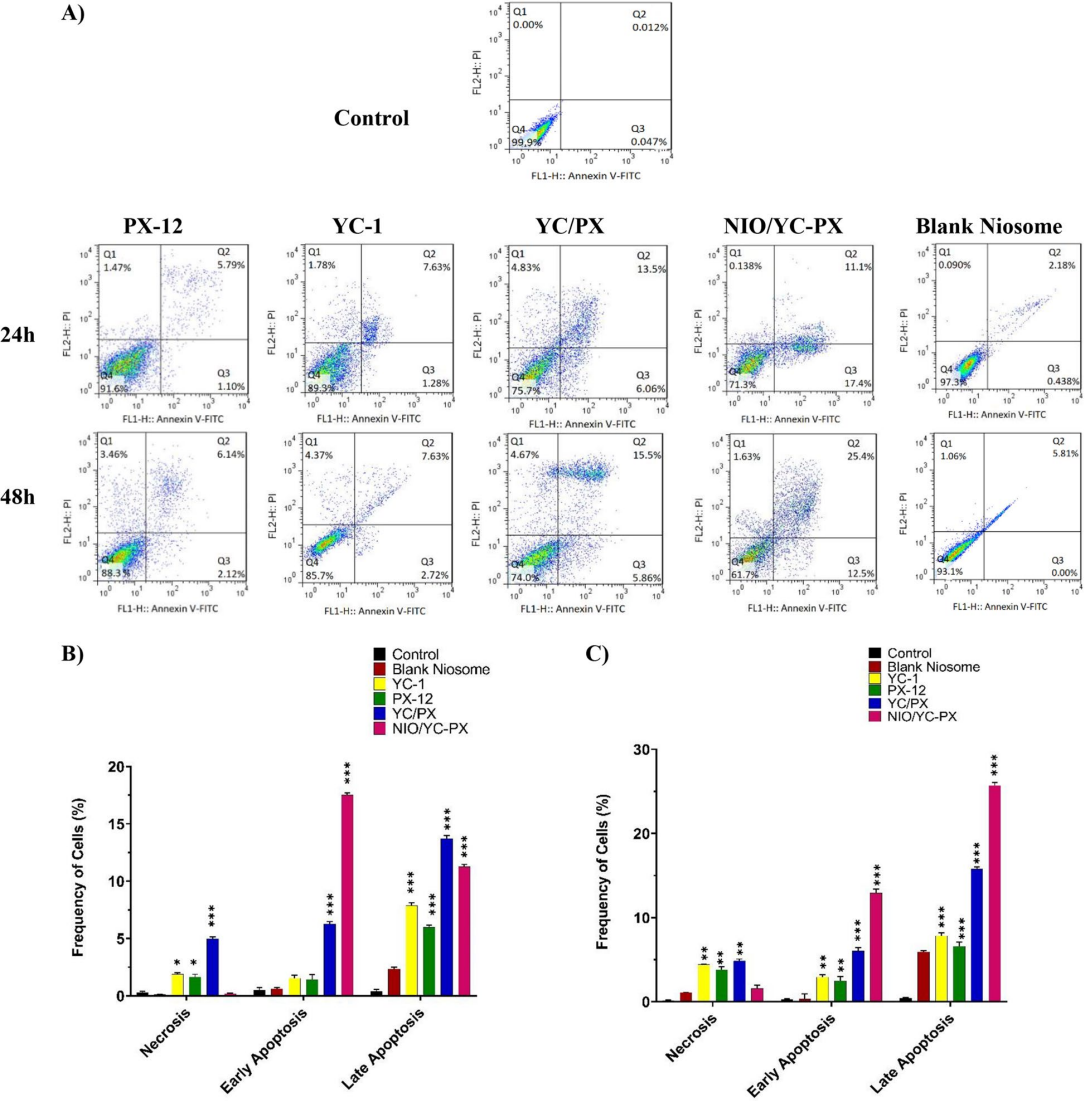
**A** Flowcytometric analysis of HT-29 cells after treatment with IC_50_ concentration of PX-12, YC-1, YC/PX, NIO/YC-PX, and free noisome. Lower left panel (Q4): live cells, upper left panel (Q1): necrosis, lower right panel (Q3): early apoptosis, upper right panel (Q2): late apoptosis. **B-C** Early and late apoptosis and necrosis of HT-29 cells treated with pX-12, YC-1, YC/PX, NIO/YC-PX, and Blank niosome after 24 and 48 h, respectively. All tests were repeated three times and the data are shown based on (mean ± SD). *p < 0.05, **p < 0.01, and ***p < 0.001

Annexin-FITC kit and flow cytometric analysis were used to evaluate the method of cell death (apoptosis or necrosis). Administration of free PX-12, free YC-1, YC/PX, NIO/YC-PX, and blank niosome to the HT-29 cells induced apoptosis in the colon cancer cell line (Fig. 8A). The percentages of apoptotic cells in HT-29 cell lines treated with blank niosome were not significantly different from the control cells that were not exposed to any of the niosomal formulations.

There were significant increases in the percentage of apoptotic HT-29 cells over the control after the colon cancer cells were exposed to NIO/YC-PX. According to these results, the rate of apoptosis (early apoptosis + late apoptosis) of drug-containing niosomes increased from %28.5 to %37.9 within 48 h. The percentages of apoptotic HT-29 cells after treatment with NIO/YC-PX increased significantly compared to those treated with freePX-12, free YC-1, and YC/PX (Fig. 8B, C). Therefore, HIF-1α inhibitors cause apoptosis of HT-29 cells. And this increase of apoptotic cells was more obvious in drugs loaded in niosome.

#### Cell cycle analysis

**Fig. 9:**
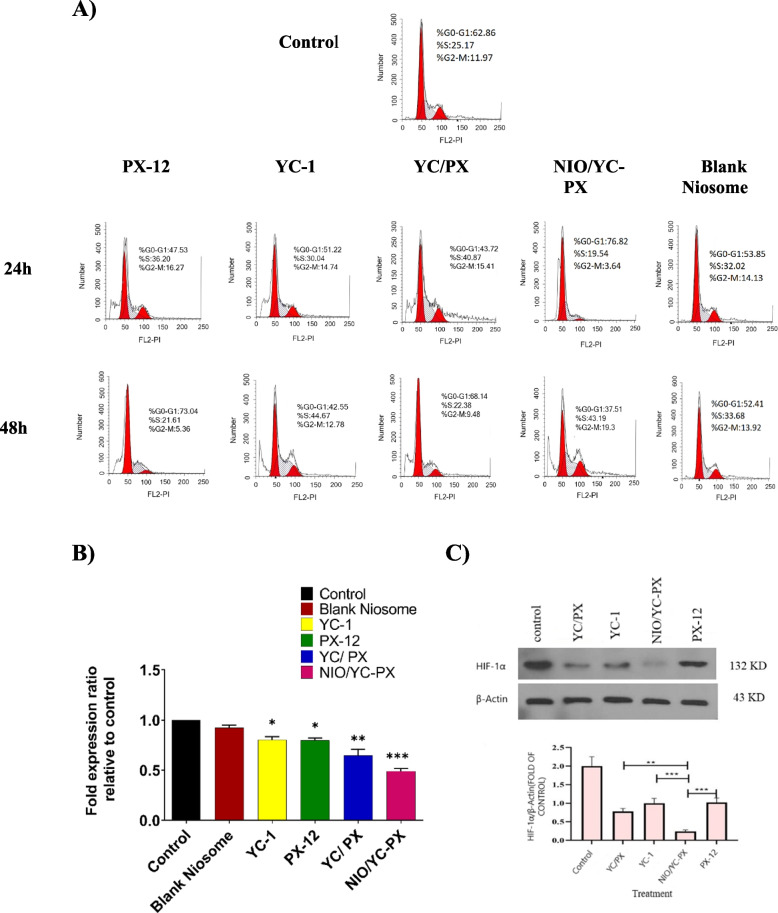
**A** The effect of free PX-12, free YC-1, YC/PX, NIO/YC-PX, and blank niosome on the cell cycle of the HT-29 cell line*.* **B** HIF-1α gene expression in HT-29 cells after treatment with PX-12, YC-1, YC/PX, NIO/YC-PX, and blank niosome at their IC_50_ values. **C** The cellular protein levels of HIF-1α were checked after treatments of HT-29 cells with YC-1, PX-12, YC/PX, and NIO/YC-PX using western blotting (up). The bands were quantified by Image J (down). All tests were repeated three times and the data are shown based on (mean ± SD). *p < 0.05, **p < 0.01, and ***p < 0.001

The effect of free PX-12, free YC-1, YC/PX, NIO/YC-PX, and blank niosome on the cell cycle of HT-29 cell line was investigated, and the results can be seen in Fig. 9A. According to the obtained results, YC-1 increases the S phase in a time interval of 48 h, but on the other hand, it decreases the G0/G1" phase. PX-12 by affecting the G0/G1 phase, increases it but decreases the S phase. By encapsulating PX-12 and Y-1 in Niosome and investigating its effect, it has been observed that the G0/G1 phase has decreased and the S phase has increased. Albeit, the results obtained from NIO/YC-PX show that over time cancer cells have accumulated in the S phase. In general, YC-1 and PX-12, both in the free state and in the niosomal formulation, cause cell cycle and cell growth arrest.

### NIO/PX-YC negatively regulates the hypoxia pathway

The inhibitory effect of drugs might be due to regulating the expression level of different genes inside the cells. The level of HIF-1α gene expression in HT-29 cells was investigated after treatment with free PX-12, free YC-1, YC/PX, NIO/YC-PX, and blank Niosome. Figure 9B shows the level of gene expression in these cells after 72 h. Based on these results, Niosome containing YC-1 and PX-12 (NIO/YC-PX) reduces the level of HIF-1α gene expression more compared to other conditions. Of course, free PX-12, free YC-1, and YC/PX also reduce the mRNA level of HIF-1α gene, while the effect of blank niosomes on gene expression is not much different compared to the control group. So, it does not have a significant effect in reducing the expression of this gene.

### Western blot analysis

To check the effect of treatments on HIF-1α protein levels, HT-29 cells were seeded and then treated with free PX-12, free YC-1, YC/PX, NIO/YC-PX then subjected to hypoxia (O_2_ < 1%) for 24 h. The protein extraction was performed, and then samples were subjected to a western blot. The results exhibited an over-expression of HIF-1α after hypoxia in the control group as compared to β-actin (Fig. 9C). But in inhibitors-treated groups, HIF-1α expression was reduced. Interestingly, in NIO/YC-PX, the HIF-1α levels were reduced significantly (Fig. 9C, down).

## Discussion

Conventional treatments for colon carcinomas (chemotherapy and surgery) have multiple side effects [56, 57], indicating the need for alternative treatment methods to increase physiological safety while increasing tumoral toxicity. One of these methods is the use of nano carriers to deliver drugs to the tumor cells in such a way that healthy cells are not affected [58]. Niosomes are one of the nanocarriers that have been suggested for drug delivery in many diseases, including cancer, span close to 20 years nowadays. One of the important features of niosomes is that they can simultaneously accommodate hydrophobic, hydrophilic, and amphiphilic molecules [59].

In this study, YC-1 (hydrophobic) and PX-12 (hydrophilic) were loaded in niosomes and investigated their anti-cancer activity on HT-29 cancer cell lines. Previous studies have shownYC-1 and PX-12 have anti-cancer activity. In a study conducted by Yangle Li et al*.,* it showed that YC-1 inhibits cell proliferation and increases apoptosis in a human bladder transitional carcinoma cell line – T24 – by inhibiting the HIF-1 pathway [60]. Also, Miau-Rong Lee and colleagues have demonstrated that YC-1 is an alternative drug to cisplatin treatment in oral cancer resistant to cisplatin, which has increased cell death and apoptosis in the CAR cell line [21]. In another study, the effect of Px-12 on colon cancer cells was investigated, which was shown to inhibit cell growth and prevent the formation of cancer cell colonies [61]. BoRaYou et al. investigated the anti-cancer activity of PX-12 on the A549 cell line (lung cancer line) and demonstrated that PX-12 inhibits the growth of A549 cells through G2/M phase arrest and ROS-dependent apoptosis [62].

In a previous study, YC-1, together with a water-soluble anti-cancer drug (Ir) that was covalently connected by succinate, were self-assembled in the form of nanoparticles. They were passively delivered to tumor tissues through increased permeability and retention effect (EPR) [63]. Up to our knowledge, YC-1 and PX-12 have not been loaded in niosomes before our study. A brief description of previous works regarding niosomes are: Abtahi et al. loaded curcumin and miR-34a simultaneously in niosomes and used them for tumor inhibition [64]; by Tanovo et al., transferrin, folic acid-conjugated doxorubicin, and curcumin-loaded niosomes were developed for delivery in breast cancer [65]; Maurer et al*.* evaluated hybrid-magnetic niosomes for siRNA delivery for breast cancer treatment [66]; El-Ridy et al*.,* designed a niosome formulation containing nystatin to increase antifungal activity and reduce toxicity against Candida albicans [67].

In our study, the effect of two variables A (lipid content) and B (surfactant:cholesterol ratio), on size, PDI, and EE% of niosome was investigated. Optimum niosomes should have the smallest size and PDI, and the highest EE%. Based on the data of the CCD method, an optimal sample is determined as A: 259.2 µmol B:1.961 molar ratioDesirability: 0.920. Our results show that the size of the particles depends on the amount of lipids, and with the increase of the value of variable A, the size of the nisome increases; on the other hand, with the increase of variable B, the size of the niosomes decreases. These results were aligned with previous studies by Ali Farmoudeh et al*.* and Priyadarshi Aparajay et al*.* [68, 69]. In addition, drug loading into noisome causes an increase in size. In our study, it has reached from 158 to 185 nm, which confirms the drug loading. With the load of drugs and the increase in size, the amount of PDI also increases (0.137 to 0.179). Based on our data, the increase of variables A and B causes a decrease in PDI. Cholesterol and surfactants play an important role in encapsulation efficiency [70]. The hydrophobicity, long alkyl chain length (C18), and the high transition temperature (50 °C) of Span 60 surfactant increase the drug %EE [71]. The encapsulation efficiency for both drugs)PX-12 and YC-1) increase with the rise of variables A and B, to an encapsulation efficiency of 91.24% for PX-12 and 78.36% for YC-1, according to our data. Furthermore, the zeta potential in this study was negative and this value became more negative with the loading of drugs in niosome (blank noisome: -4.56 and NIO/Px-Yc: -7.10). The negative zeta potential may be related to the hydroxyl groups in cholesterol [72]. Improper zeta potential leads to the accumulation of vesicles, toxicity and lack of specificity in targeting [68].

In addition to determining the morphology of niosomes, the size of these particles was also measured by TEM. Based on our results, the determined size is smaller than the results obtained from DLS, which may be due to the difference in TEM and DLS techniques.SEM and TEM provide the size of the nanoparticles as dried (the exact diameter of each particle), while DLS measures the hydrodynamic diameter, which includes the core along with any molecules bound or adsorbed onto the surface, such as ions and water molecules [73].

The stability results show that niosomes are more stable at 4 ˚C than 25 ˚C. The size, PDI and EE% of niosomes changed less after 60 days at 4 ˚C than at 25 ˚C. The hydrophobic part of niosomes is stronger and harder at lower temperatures, which is probably due to the accumulation and integration of it. The reason for the increase in size at room temperature may be due to the swelling of the niosomes and the hydrogel around them. Encapsulation efficiency decreases at room temperature, which is probably due to removal from the niosome surface and increased fluidity of lipid vesicles [74]. With regard to drug release, our data shows that the rate of release depends on the pH of the environment. Based on our results at pH 5.4 (acidic and closer to a cancer environment), the release rate of drugs from niosomes is higher than at pH 7.4 [58].

Taken the previous data collectively, our niosome was used in the optimal form after synthesis for cellular and molecular tests on the HT-29 cell line. Although few researchers reported very different rates of particle uptake in some cancer cell lines. Slight particle size, surface charge differences, and tissues where cell lines originate had significant implications in the cellular uptake of NPs, and various mechanisms were involved in the uptake process. As an example In vivo bio distribution suggested that NPs with slight negative charges and particle size of 150 nm were tended to accumulate in tumor more efficiently [75] which is near to our nanoniosome size and charge (Sect. 3.3). Also, our cytotoxicity and molecular findings after treatment of cells with drug loaded niosomes as compared to control groups verified proper cell uptake of NIO/PX-YC.

The results of the MTT test show that cell death caused by NIO/PX-YC is more than other cases, and this effect increases with time. As illustrated in Fig. 9, in the HT-29 cell line, the survival rate of NIO/PX- YC with a concentration of 25 µmol (within 72 h) has decreased from approximately 60% to about 40%. Also, with the increase of the concentration (from 1.56 µM to 25 µM), the survival rate decreased in all cases. Therefore, cell survival depends on both time and concentration. The blank niosome has shown acceptable toxicity and does not have a significant effect on reducing the survival rate. The results obtained from HFF cell line show that, compared to HT-29 cells, NIO/PX-YC has lower toxicity and higher survival rate.Annexin/PI staining and histogram analysis proves the apoptotic property of drug-containing niosomes, which was accompanied by an increase in dead cells in the subG1 phases of NIO/PX-YC-induced apoptosis in HT-29 cells. Our data on cell cycle analysis shows that YC-1 causes colon cancer cells to accumulate in the S phase and arrest the G0/G1 phase. Similar results have been reported in the HCT116 cell line, human hepatocellular carcinoma and human umbilical vein endothelial cells [76]. Here, Px-12 increases the accumulation of cells in the G0/G1 phase and arrest on the S phase in the HT-29 cell line, data in alignment with the DLD-1 and SW620 cell lines where reduction and arrest of cells in S phase was demonstrated [61]. Albeit, the results obtained from NIO/Px-Yc show that over time cancer cells have accumulated in S phase. In general, YC-1 and PX-12 both in the free state and in the niosomal formulation, cause cell cycle and cell growth arrest.

A known target for both YC-1 and PX-12 is the hypoxia pathway, particularly relevant for solid tumor survival, involving neoangiogenesis through VEGF-dependent signaling [17, 19, 25, 26]. HIF-1α gene expression analysis demonstrates that NIO/PX-YC have a significant effect on HIF-1α transcript levels. Expectedly, free PX-12, free YC-1, and PX-12 & YC-1 combination also decrease the mRNA levels of HIF-1α gene, while the empty niosome has no significant effect on reducing this gene’s expression. Attheproteinlevel, YC-1 has been shown to decrease the expression of HIF-1α protein in hypoxic conditions, yet with a greater effect in normoxia [77], while PX-12 has been shown to reduce HIF-1α protein levels predominantly in hypoxia [78]. Our findings demonstrate that the combination of the two drugs further decreased the level of HIF-1α protein, an effect that was further significantly reduced by loading the two drugs into our niosomes. No decrease in HIF-1α protein level was observed in blank niosomes. As mentioned, loading substances and drugs into niosomes increases their effectiveness [79], and collectively our niosome-loaded drugs increased the inhibitory effect compared to the free state. Consequently, the reduced expression of HIF-1α both at the mRNA and protein levels by our NIO/PX-YC is expected to decrease HIF-mediated vascular and metabolic genes (e.g., VEGF and the proton pump carbonic anhydrase 9), thus contributing to reduced angiogenesis and acidification of the tumoral milieu putatively leading to cancer cells’ growth arrest while further improving the delivery of drugs loaded into niosomes. Nevertheless, despite expected and even predictable as previously demonstrated in vitro [69, 70], studies in in vivo models of solid tumors will be necessary to further corroborate this hypothesis.

## Conclusion

HIF-1α is among the key factors responsible for CRC progression, anti-apoptosis, and tumor-supporting phenomena. Targeting of HIF-1α may become more successful when a combination of two inhibitors co-loaded in nanodelivery niosome system. In the present work, the hydrophobic YC-1 and the hydrophilic PX-12 as HIF-1α inhibitors were co-loaded in corresponding locations in the niosomes structure. The behavior of their release from niosome shows a stable release compared to the free forms. The cell tests that were conducted collectively proved that YC-1 and PX-12 do not have significant toxicity in their free form, but when they are co-loaded into the niosome, remarkably, a considerable decrease in the level of HIF-1α at mRNA and protein levels is observed. Also, their toxicity increases and causes apoptosis and cell cycle arrest. Taken together, the co-loading of niosomes with YC-1 and PX-12 can be a promising strategy for the controllable, stable, and effective inhibition of HIF-1α in CRC.

### Supplementary Information

**Additional file 1:** **Table S1.** Variance analysis of the quadratic polynomial model for size. **Table S2. **Summary of regression analysis results for response size by fitting in the quadratic model. **Table S3.** Variance analysis of the quadratic polynomial model for PDI. **Table S4. **Summary of results of regression analysis for responses PDI, for fitting to the quadratic model. **Table S5**. Variance analysis of the quadratic polynomial model for EE (PX, YC). **Table S6. **Summary of regression analysis results for response EE (PX, YC) by fitting in the quadratic model. **Table S7.** The kinetic release models and the parameters obtained for optimum niosomal formulation.

## Data Availability

The data will be available upon request.
