# Supplementary material for: Efficient targeting of HIF-1α mediated by YC-1 and PX-12 encapsulated niosomes: potential application in colon cancer therapy
Source: J Biol Eng. 2023 Sep 25;17:58. doi: 10.1186/s13036-023-00375-3 (PMC10521571; doi:10.1186/s13036-023-00375-3)
Supplement: Supplementary file 1 — Additional file 1: Table S1. Variance analysis of the quadratic polynomial model for size. Table S2. Summary of regression analysis results for response size by fitting in the quadratic model. Table S3. Variance analysis of the quadratic polynomial model for PDI. Table S4. Summary of results of regression analysis for responses PDI, for fitting to the quadratic model. Table S5. Variance analysis of the quadratic polynomial model for EE (PX, YC). Table S6. Summary of regression analysis results for response EE (PX, YC) by fitting in the quadratic model. Table S7. The kinetic release models and the parameters obtained for optimum niosomal formulation. [file 13036_2023_375_MOESM1_ESM.docx]

*Supplementry data for*

**Efficient Targeting of HIF-1α Mediated by YC-1 and PX-12 Encapsulated Niosomes: Potential Application in Colon Cancer Therapy**

**Azar Bakand^1†^, Sevil Vaghefi Moghaddam^2†^, Maryam Naseroleslami^3^, Helder André^4^, Neda Mousavi-Niri*^5^, and Effat Alizadeh^1*^**

^1^Department of Medical Biotechnology, Faculty of Advanced Medical Sciences, Tabriz University of Medical Sciences, Tabriz, Iran.

^2^Clinical Research Development, Unit of Tabriz Valiasr Hospital, Tabriz University of Medical Sciences, Tabriz, Iran.

^3^Department of Cellular and Molecular Biology, Faculty of Advanced Science and Technology, Tehran Medical Sciences, Islamic Azad University, Tehran, Iran.

^4^Department of Clinical Neuroscience, St. Erik Eye Hospital, Karolinska Institute, 11282 Stockholm, Sweden.

^5^Department of Biotechnology, Faculty of Advanced Science and Technology, Tehran Medical Sciences, Islamic Azad University, Tehran, Iran.

***Corresponding authors:**

*****Effat Alizadeh: Department of Medical Biotechnology, Faculty of Advanced Medical Sciences, Tabriz University of Medical Sciences, Tabriz, Iran, [e.alizadeh.2010@gmail.com](mailto:e.alizadeh.2010@gmail.com).

*****NedaMousavi-Niri: Department of Biotechnology, Faculty of Advanced Science and Technology, Tehran Medical Sciences, Islamic Azad University, Tehran, Iran. [neda.mousaviniri@gmail.com](mailto:neda.mousaviniri@gmail.com), [n.mousavi@iautmu.ac.ir](mailto:n.mousavi@iautmu.ac.ir) .

**†** These authors have contributed equally to this work.

**Table S1**. Variance analysis of the quadratic polynomial model for size.

| Source | Sum of squares | Degree of Freedom | Mean square | F-Value | P-Value | Evaluation |
| --- | --- | --- | --- | --- | --- | --- |
| Model | 22095.77 | 5 | 4419.15 | 38.76 | 0.0005 | significant |
| A | 2242.67 | 1 | 2242.67 | 19.67 | 0.0068 |  |
| B | 10110.61 | 1 | 10110.61 | 88.68 | 0.0002 |  |
| AB | 1564.20 | 1 | 1564.20 | 13.72 | 0.0139 |  |
| A^2^ | 2163.14 | 1 | 2163.14 | 18.97 | 0.0073 |  |
| B^2^ | 3887.07 | 1 | 3887.07 | 34.09 | 0.0021 |  |

**Table S2.** Summary of regression analysis results for response size by fitting in the quadratic model.

| Quadratic model | R^2^ | Adjusted R^2^ | Adeq Precision | Lack of fit |
| --- | --- | --- | --- | --- |
|  | 0.9748 | 0.9497 | 15.426 | 0.1761 |
| Size = +192.63 + 19.33 × A - 41.05 × B + 19.77 × AB + 29.22 × A^2^ + 39.17 × B^2^ | | | | |

**Table S3**. Variance analysis of the quadratic polynomial model for PDI.

| Source | Sum of squares | Degree of Freedom | Mean square | F-Value | P-Value | Evaluation |
| --- | --- | --- | --- | --- | --- | --- |
| Model | 0.018 | 5 | 3.531E-003 | 20.18 | 0.0025 | significant |
| A | 3.682E-004 | 1 | 3.682E-004 | 2.10 | 0.2066 |  |
| B | 0.013 | 1 | 0.013 | 74.15 | 0.0003 |  |
| AB | 1.980E-003 | 1 | 1.980E-003 | 11.32 | 0.0200 |  |
| A^2^ | 9.019E-004 | 1 | 9.019E-004 | 5.15 | 0.0724 |  |
| B^2^ | 8.088E-004 | 1 | 8.088E-004 | 4.62 | 0.0842 |  |

**Table S4.** Summary of results of regression analysis for responses PDI, for fitting to the quadratic model.

| Quadratic model | R^2^ | Adjusted R^2^ | Adeq Precision | Lack of fit |
| --- | --- | --- | --- | --- |
|  | 0.9528 | 0.9056 | 14.530 | 0.3494 |
| PDI = +0.21 - 7.833E-003 × A – 0.046 × B + 0.022 × AB + 0.019 × A^2^ + 0.018 × B^2^ | | | | |

**Table S5**. Variance analysis of the quadratic polynomial model for EE (PX, YC).

| Source | Sum of squares | | Degree of Freedom | Mean square | | F-Value | | | | P-Value | | | Evaluation | |  |
| --- | --- | --- | --- | --- | --- | --- | --- | --- | --- | --- | --- | --- | --- | --- | --- |
|  | PX | YC |  | PX | YC | | PX | YC | | | PX | YC | |  | |
| Model | 331.34 | 511.07 | 5 | 66.27 | 102.21 | | 8.89 | | 7.16 | | 0.0158 | 0.0249 | | significant | |
| A | 198.15 | 22.70 | 1 | 198.15 | 22.70 | | 26.58 | | 1.59 | | 0.0036 | 0.2630 | |  | |
| B | 97.61 | 439.64 | 1 | 97.61 | 439.64 | | 13.09 | | 30.79 | | 0.0152 | 0.0026 | |  | |
| AB | 4.75 | 5.55 | 1 | 4.75 | 5.55 | | 0.64 | | 0.39 | | 0.4609 | 0.5605 | |  | |
| A^2^ | 26.34 | 39.05 | 1 | 26.34 | 39.05 | | 3.53 | | 2.73 | | 0.1190 | 0.1591 | |  | |
| B^2^ | 0.46 | 13.16 | 1 | 0.46 | 13.16 | | 0.061 | | 0.92 | | 0.8145 | 0.3812 | |  | |

**Table S6.** Summary of regression analysis results for response EE (PX, YC) by fitting in the quadratic model.

| Quadratic model | R^2^ | Adjusted R^2^ | Adeq Precision | Lack of fit |
| --- | --- | --- | --- | --- |
| PX | 0.8989 | 0.7977 | 9.700 | 0.2074 |
| YC | 0.8774 | 0.7549 | 8.660 | 0.0990 |
| EE (PX) = +87.90+5.75× A +4.03× B -1.09 × AB -3.22× A^2^-0.42× B^2^ | | | | |
| EE (YC) = +65.07+1.95 × A +8.56× B -1.18 × AB -3.93× A^2^+2.28× B^2^ | | | | |

**Table S7.** The kinetic release models and the parameters obtained for optimum niosomal formulation.

| Release Model | Zero-Order | Korsmeyer-Peppas | | First-Order | Higuchi |
| --- | --- | --- | --- | --- | --- |
|  | R^2^ | R^2^ | n* | R^2^ | R^2^ |
| PX (aq) | 0.4483 | 0.7425 | 0.3900 | 0.7478 | 0.6125 |
| YC (aq) | 0.4807 | 0.7476 | 0.4361 | 0.9198 | 0.6534 |
| PX (7.4) | 0.8138 | 0.9726 | 0.4373 | 0.8902 | 0.9376 |
| YC (7.4) | 0.8116 | 0.9599 | 0.4823 | 0.8661 | 0.9357 |
| PX (5.4) | 0.8205 | 0.9463 | 0.3185 | 0.5965 | -0.3185 |
| YC (5.4) | 0.7974 | 0.9085 | 0.5674 | 0.5317 | 0.9140 |

* Diffusion or release exponent
